# Supplementary material for: Genetic Characterization of Salmonella Infantis with Multiple Drug Resistance Profiles Isolated from a Poultry-Farm in Chile
Source: Microorganisms. 2021 Nov 17;9(11):2370. doi: 10.3390/microorganisms9112370 (PMC8621671; doi:10.3390/microorganisms9112370)
Supplement: Supplementary file 1 [file microorganisms-09-02370-s001.zip › Supplementary_Fig_S1.pdf]

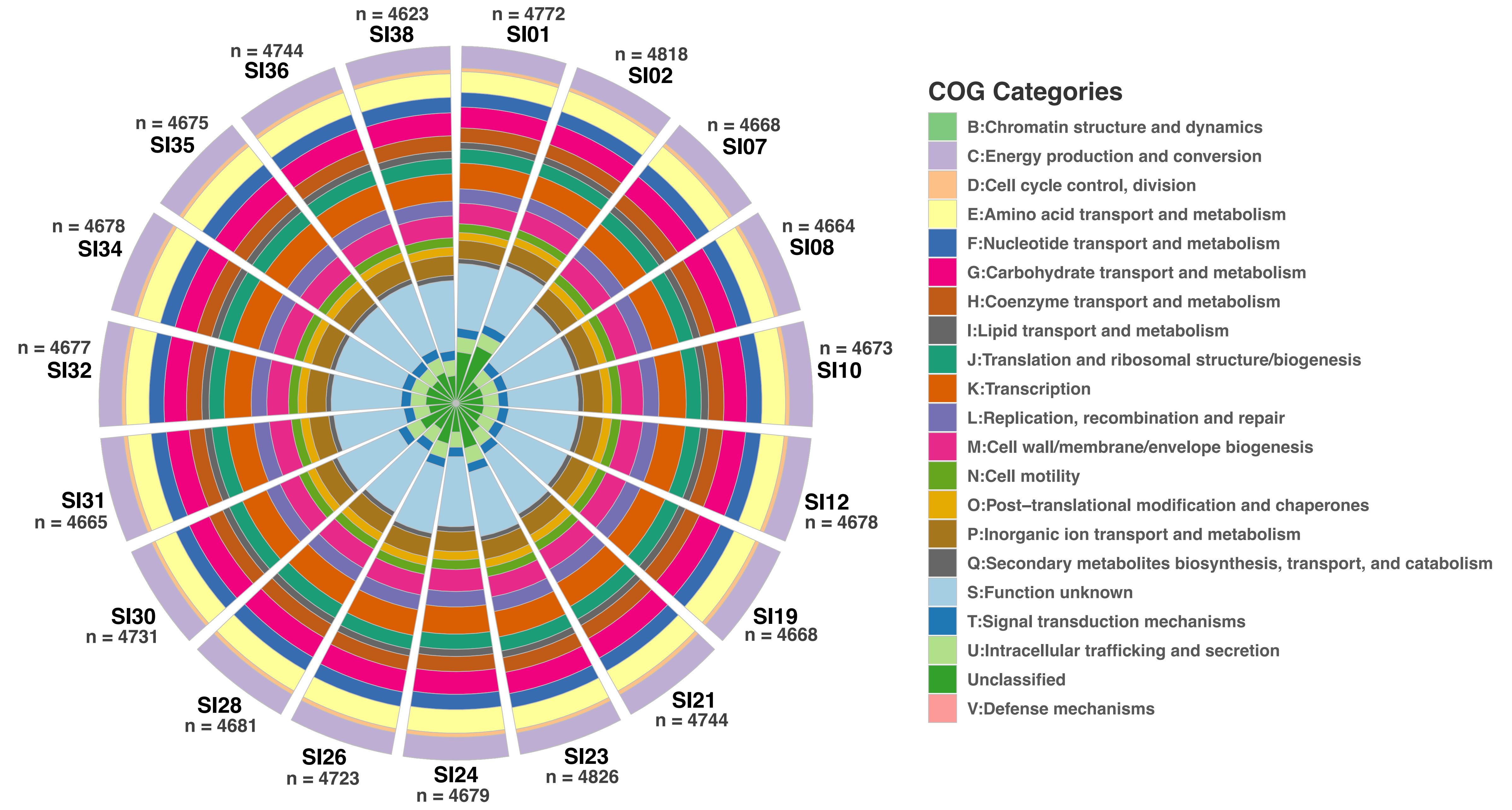
n = 4623
SI38
n = 4772
SI01
n = 4818
SI02
n = 4668
SI07
n = 4664
SI08
n = 4673
SI10
SI12
n = 4678
SI19
n = 4668
SI21
n = 4744
SI23
n = 4826
SI24
n = 4679
SI26
n = 4723
SI28
n = 4681
SI30
n = 4731
SI31
n = 4665
n = 4677
SI32
n = 4678
SI34
n = 4675
SI35
n = 4744
SI36
COG Categories
B:Chromatin structure and dynamics
C:Energy production and conversion
D:Cell cycle control, division
E:Amino acid transport and metabolism
F:Nucleotide transport and metabolism
G:Carbohydrate transport and metabolism
H:Coenzyme transport and metabolism
I:Lipid transport and metabolism
J:Translation and ribosomal structure/biogenesis
K:Transcription
L:Replication, recombination and repair
M:Cell wall/membrane/envelope biogenesis
N:Cell motility
O:Post–translational modification and chaperones
P:Inorganic ion transport and metabolism
Q:Secondary metabolites biosynthesis, transport, and catabolism
S:Function unknown
T:Signal transduction mechanisms
U:Intracellular trafficking and secretion
Unclassified
V:Defense mechanisms
