# Supplementary figures and images for: Genetic Characterization of Salmonella Infantis with Multiple Drug Resistance Profiles Isolated from a Poultry-Farm in Chile
Source: Microorganisms. 2021 Nov 17;9(11):2370. doi: 10.3390/microorganisms9112370 (PMC8621671; doi:10.3390/microorganisms9112370)

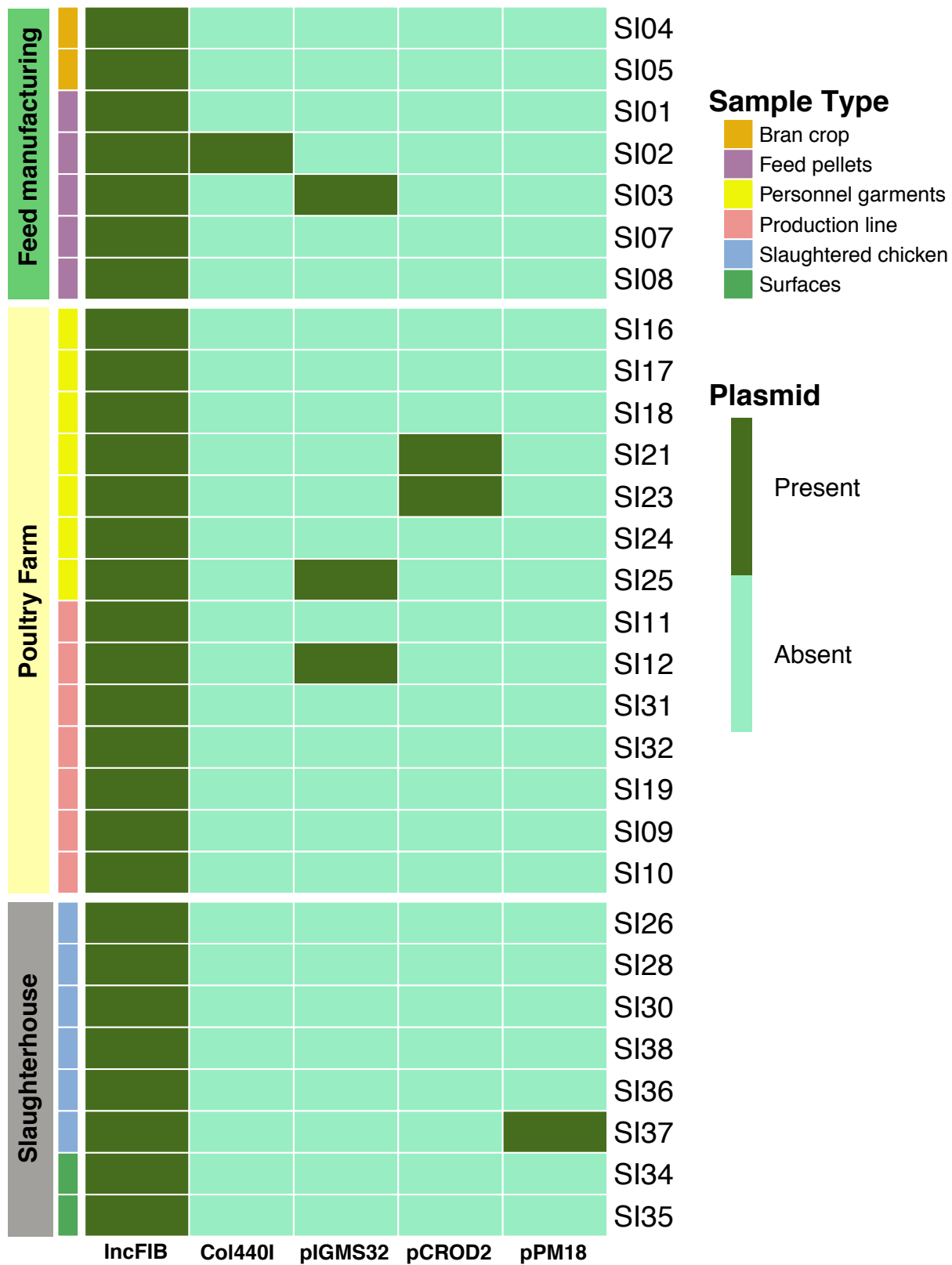

Supplement: Supplementary file 1 [file microorganisms-09-02370-s001.zip › Supplementary_Fig_S2.pdf]
